# The Quinonoid Zwitterion Interlayer for the Improvement of Charge Carrier Mobility in Organic Field-Effect Transistors

**DOI:** 10.3390/polym13101567

**Published:** 2021-05-13

**Authors:** Adam Luczak, Angélina Torres Ruiz, Simon Pascal, Adrian Adamski, Jarosław Jung, Beata Luszczynska, Olivier Siri

**Affiliations:** 1 Department of Molecular Physics, Faculty of Chemistry, Lodz University of Technology, Zeromskiego 116, 90-924 Lodz, Poland; adam.luczak@p.lodz.pl (A.L.); adrian.adamski@pwr.edu.pl (A.A.); jaroslaw.jung@p.lodz.pl (J.J.); 2 CNRS, CINaM, UMR 7325, Campus de Luminy, Aix Marseille Université, CEDEX 09, 13288 Marseille, France; angelina.torres-ruiz@etu.univ-amu.fr (A.T.R.); pascal@cinam.univ-mrs.fr (S.P.); 3 Department of Experimental Physics, Faculty of Fundamental Problems of Technology, Wroclaw University of Science and Technology, Wyb. Wyspianskiego 27, 50-370 Wroclaw, Poland

**Keywords:** zwitterionic benzoquinonemonoimine, organic field-effect transistors, interface, charge carrier trapping, electrostatic force microscopy

## Abstract

The interface between the semiconductor and the dielectric layer plays a crucial role in organic field-effect transistors (OFETs) because it is at the interface that charge carriers are accumulated and transported. In this study, four zwitterionic benzoquinonemonoimine dyes featuring alkyl and aryl *N*-substituents were used to cover the dielectric layers in OFET structures. The best interlayer material, containing aliphatic side groups, increased charge carrier mobility in the measured systems. This improvement can be explained by the reduction in the number of the charge carrier trapping sites at the dielectric active layer interface from 10^14^ eV^−1^ cm^−2^ to 2 × 10^13^ eV^−1^ cm^−2^. The density of the traps was one order of magnitude lower compared to the unmodified transistors. This resulted in an increase in charge carrier mobility in the tested poly [2,5-(2-octyldodecyl)-3,6-diketopyrrolopyrrole-alt-5,5-(2,5-di(thien-2-yl)thieno [3,2-b]thiophene)] (DPPDTT)-based transistors to 5.4 × 10^−1^ cm^2^ V^−1^ s^−1^.

## 1. Introduction

The interface between semiconductors and dielectrics has attracted much attention due to its effects on the morphology of organic semiconductors (OSCs) and charge transport in organic field-effect transistors (OFETs) [1]. Several interfacial factors have been identified as influencing the performance of OFETs [2]. Important parameters for the semiconductor–insulator interface include insulator surface roughness, surface energy, surface polarity, and the dielectric constant [3]. These parameters can combine to produce several effects, such as changes to the morphological structure of the semiconductor thin film and improved charge transport performance, with strong dependence on the hydrophobicity of the substrate surface. OSCs can be deposited on the dielectric surface by different technological techniques such as vacuum evaporation or coating from solution. The properties of the deposited layer strictly depend on the molecular stacking of OSC molecules. The most favorable stacking of OSC involves face-to-face π-π interactions [4], which generate the shortest intermolecular distances and very good π-electron delocalization. This enables improved charge carrier transport between molecules by hopping [5]. Such arrangements are obtained when the dielectric surface has hydrophobic properties. The presence of alkyl chains borne by OSC molecules implies arrangements in which the long axes of the OSC molecules are parallel to the substrate surface. Due to its hydrophobic nature, an OSC–modified dielectric interface may inhibit protonation of the siloxyl groups on the SiO_2_ dielectric surface, resulting in better performance and improved stability of the channel in the OFET [6]. However, most metal oxide dielectric surfaces (e.g., SiO_2_) are hydrophilic [6], and for that reason the surface of the dielectric has to be tailored to match the hydrophobicity of the OSC.

Most materials used for surface modification in OFET fabrication are based on organosilicon derivatives such as hexamethyldisilazane (HMDS) or octadecyltrimethoxysilane (OTS) [7]. These materials are able to increase the hydrophobicity at the OSC–metal oxide interface. For example, the water contact angle can be increased to 110° at a surface modified with HMDS [6]. However, the protocol for such modification requires the use of high temperatures, oxygen plasma treatment, or piranha acid (i.e., an aqueous H_2_SO_4_/H_2_O_2_ solution). In this context, the quality of the semiconductor–gate dielectric interface is critical for the performance of OFETs [8,9,10]. Each disorder of the electric field strength on the above gate dielectric can create charge traps or additional charge carriers, depending on the character of the irregularity [10]. The performance of OFETs is strongly influenced by the polarity of the dielectric surfaces. The presence of higher polar surfaces may cause lower charge carrier mobility, and hysteresis is seen in the I–V characteristics [9]. The presence of defects at the interface is visible in the current–voltage curves as any of the following: (i) hysteresis, which is the difference between the curves measured with increased and decreased drain voltage [11]; (ii) a high OFF current value; or (iii) an ON current smaller than those described in the literature [12].

To prevent hysteresis and provide good charge transport properties in thin OSC films, the electric field at the interface has to be monotonically variable along the transistor channel. Thus, the deposition of a semiconductor–dielectric interlayer is necessary to improve the quality of the transistor. As already mentioned, the most popular form of dielectric surface modification consists of the deposition of an ultrathin layer of an organosilicon compound such as HMDS or OTS [5,13,14]. Previous studies show that the existence of poor interface contacts and energy losses in devices can be overcome by the use of zwitterions as an interlayer to improve performance [15,16,17,18,19]. The remarkable properties of zwitterions can be explained by their large intrinsic dipole moment. They are also soluble in orthogonal solvents, which means that a previously deposited zwitterion-based layer will not be dissolved during the deposition of a semiconducting layer using spin-coating methods.

**Scheme 1:**
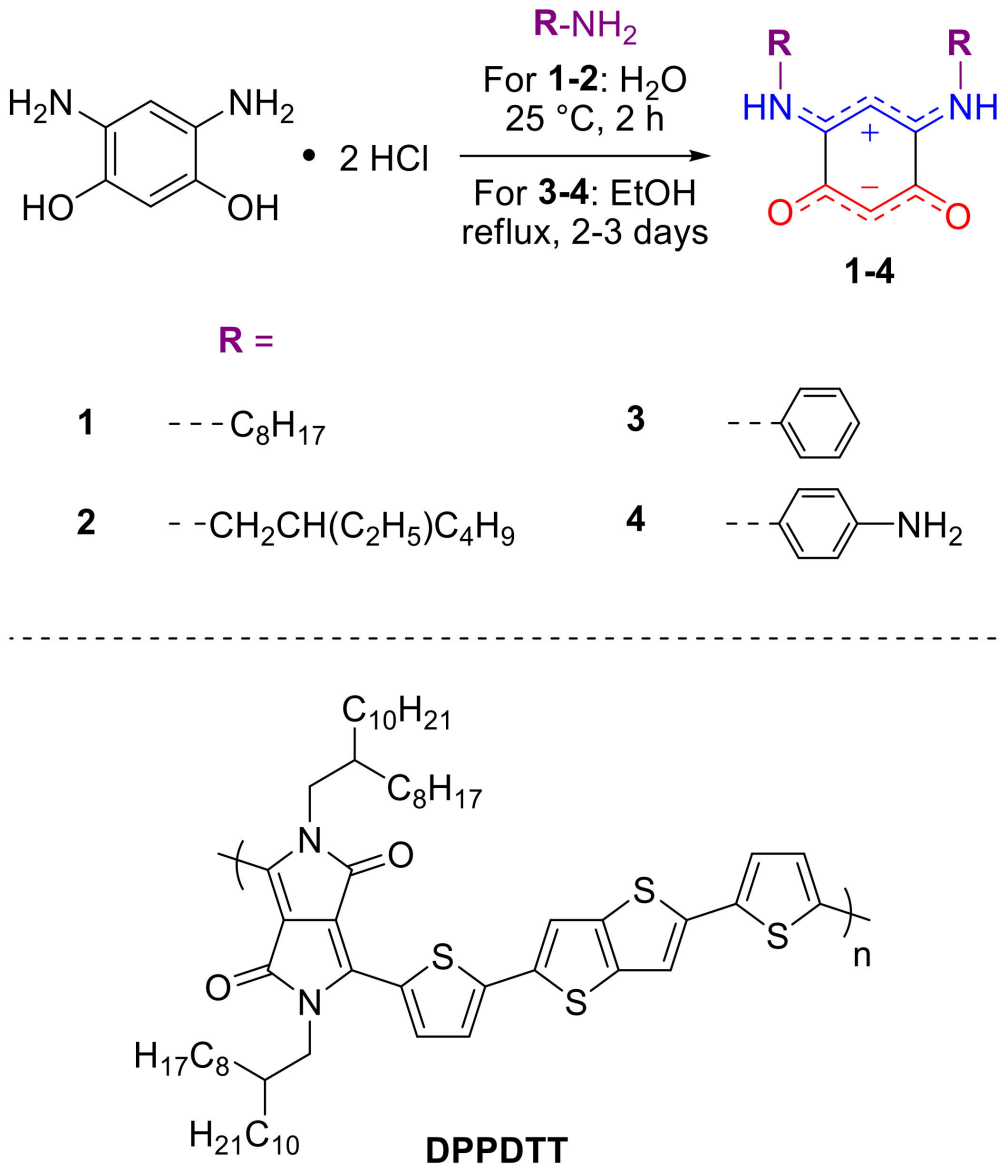
A series of zwitterionic BQMIs (**1–4**) used as modifiers of the dielectric surface, and the schematic structure of the DPPDTT organic semiconductor.

Here, we propose an innovative approach, whereby an ultrathin layer of organic dipolar molecules endowed with a large intrinsic dipole (ca. 10 D) is deposited between the silicon wafer and the OSC layer. We decided to investigate zwitterionic benzoquinonemonoimine (BQMI) dyes **1–4** (Scheme 1), which have recently been proposed for the creation of molecular films following adsorption [20,21,22,23,24], the polymerization of metallic surfaces [25,26,27], for use in interfaces [28,29], and, more recently, as interlayers in graphene transistors [30]. Lin et al. reported a zwitterion-based cathode interlayer in organic photovoltaics, showing that the introduction of such interlayer significantly suppresses trap-assisted recombination in photovoltaics. Moreover, the efficiency of the device was fairly insensitive to the interlayer thickness, which could be beneficial for mass-production technologies such as ink-jet printing or screen printing [31]. The variation of the chemical functions of these BQMI dyes (i.e., the modification of the N-substituents) by the transamination reaction [32] provides an opportunity to tune the degree of hydrophobicity and to create a smooth and homogeneous charge on the dielectric surface. However, there are no reports in the literature on their use as interlayer components to improve the quality of the semiconductor–dielectric interface in OFETs.

## 2. Materials and Methods

### 2.1. Materials and Synthesis

The organic semiconductor poly [2,5-(2-octyldodecyl)-3,6-diketopyrrolopyrrole-alt-5,5-(2,5-di(thien-2-yl)thieno [3,2-b]thiophene)] (abbreviated as DPPDTT), characterized by a molecular weight (MW) of 290.668 kDa and a polydispersity index (PDI) of 2.03, was purchased from ABCompany GmbH (Riedering, Germany). Silicon wafers were procured from the same source. Solvents (HPLC grade) were provided by Sigma-Aldrich (Merck KGaA, Darmstadt, Germany). All compounds and solvents were used as received, without additional treatment, except for compounds **3–4,** which were prepared according to procedures described elsewhere [33,34]. The synthesis of all compounds (**1–4**) is described below, starting from 4,6-diaminoresorcinol dihydrochloride (Sigma-Aldrich) or unsubstituted BQMI zwitterions. Melting points (M.P.) were measured in open capillary tubes using a STUART SMP30 melting points apparatus and are presented without correction.

### 2.2. Synthesis of Compound ***1***

4,6-Diaminoresorcinol dihydrochloride (500 mg, 1.76 mmol, 1 equiv.) was dissolved in 10 mL of distilled water. Next, *n*-octylamine (2.33 mL, 14.08 mmol, 10 equiv.) was added and the solution was stirred in the presence of air at room temperature for 2 h. The mixture was extracted with dichloromethane and the organic layer was washed twice with distilled water, dried over anhydrous Na_2_SO_4_, filtered, and evaporated under reduced pressure. The residue was then triturated in pentane, filtered on sintered glass, and finally dried under a vacuum to afford product **1** as a pink solid (358 mg, 73% yield). M.P.: 129 °C. ^1^H NMR (CDCl_3_, 400 MHz): δ = 8.24 (br s, 2H, NH), 5.45 (s, 1H, CH), 5.12 (s, 1H, CH), 3.35 (m, 4H, NCH_2_), 1.73 (quint, *^3^J* = 7.6 Hz, 4H, CH_2_), 1.31–1.27 (m, 20H, CH_2_), 0.88 (t, *^3^J* = 7.0 Hz, 6H, CH_3_). ^13^C{^1^H} NMR (CDCl_3_, 100 MHz): δ = 172.5 (C), 156.7 (C), 99.0 (CH), 80.7 (CH), 43.4 (NCH_2_), 31.9 (CH_2_), 29.2 (CH_2_), 29.2 (CH_2_), 28.4 (CH_2_), 27.1 (CH_2_), 22.7 (CH_2_), 14.2 (CH_3_). IR (neat, cm^−1^): ν = 3166, 2953, 2920, 2853, 1642, 1528, 1472, 1368, 1290, 1263, 1179, 949, 857, 822, 780, 724. HRMS (ESI+): *m/z* calculated for [M+H]^+^: 363.3006 (C_22_H_39_N_2_O_2_^+^), found: 363.3002.

### 2.3. Synthesis of Compound ***2***

4,6-Diaminoresorcinol dihydrochloride (500 mg, 2.35 mmol, 1 equiv.) and 2-ethylhexylamine (2.32 mL, 23.47 mmol, 10 equiv.) were dissolved in water (10 mL). The reaction was stirred for 2 h at room temperature. The crude mixture was diluted with dichloromethane, washed with water, and then dried over anhydrous Na2SO4 and concentrated. The resulting solid was purified via dissolution in a minimum amount of dichloromethane, followed by precipitation in pentane and filtration on sintered glass. The powder was rinsed several times with pentane and finally dried under a vacuum to afford product **2** as a purple solid (534 mg, 63% yield). M.P.: 171 °C. ^1^H NMR (400 MHz, CDCl_3_): δ = 8.28 (s, 2H, NH), 5.46 (s, 1H, CH), 5.14 (s, 1H, CH), 3.27 (t, *^3^J* = 6.1 Hz, 4H, NCH_2_) 1.68 (m, 2H, CH) 1.44–1.31 (m, 16H, CH_2_), 0.95–0.89 (m, 12H, CH_3_). ^13^C{^1^H} NMR (CDCl3, 100 MHz): δ = 172.4 (C), 157.0 (C), 98.8 (CH), 80.7 (CH), 46.5 (NCH2), 39.1 (CH), 31.2 (CH_2_), 29.0 (CH_2_), 24.5 (CH_2_), 23.0 (CH_2_), 14.2 (CH_3_), 11.0 (CH_3_). IR (neat, cm^−1^): ν = 3168, 2956, 2924, 2860, 1642, 1530, 1478, 1451, 1377, 1352, 1301, 1233, 1126, 1060, 954, 851, 822, 781, 732. HRMS (ESI+): *m/z* calculated for [M+H]^+^: 363.3006 (C_22_H_39_N_2_O_2_^+^), found: 363.3005.

### 2.4. Synthesis of Compound ***3***

Unsubstituted BQMI (250 mg, 1.81 mmol, 1 equiv.) and aniline (8.43 g, 90.5 mmol, 50 equiv.) were dissolved in absolute ethanol (25 mL). The reaction mixture was heated to reflux for 3 days and the solvent was evaporated under reduced pressure. Et_2_O was added to the reaction mixture. The precipitate was filtered on sintered glass then rinsed with Et_2_O. The filtrate was put aside, and the remaining precipitate was rinsed with acetone, DCM, and MeOH. The resulting filtrate was evaporated to afford product **3** as a brown solid (128 mg, 24% yield). M.P: 260 °C. ^1^H NMR (400 MHz, DMSO-d*6*): δ = 10.97 (br s, 2H, NH), 7.39 (m, 8H, CH_Ar_), 7.29 (t, *^3^J* = 7.1 Hz, 2H, CH_Ar_), 5.81 (s, 1H, CH), 5.20 (s, 1H, CH).^13^C{^1^H} NMR (101 MHz, DMSO-d*6*): δ = 171.9 (C_quat_), 155.5 (C_quat_), 136.5 (C_quat_), 129.2 (CH), 127.5 (CH), 124.9 (CH), 98.2 (CH), 85.2 (CH). IR (neat, cm^−1^): ν = 3286, 3043, 2119, 2088, 2007, 1957, 1722, 1637, 1576, 1473, 1437, 1392, 1356, 1286, 1232, 1176, 1107, 1022, 972, 921, 854, 796, 750, 692. HRMS (ESI+): *m/z* calculated for [M+H^+^]: 291.1128 (C_18_H_14_N_2_O_2_^+^), found: 291.1129.

### 2.5. Synthesis of Compound ***4***

Under an inert atmosphere, 1,4-phenylenediamine dihydrochloride (11.86 g, 65.16 mmol, 30 equiv.) and DIPEA (22.55 mL, 130.32 mmol, 60 equiv.) were added to a suspension of unsubstituted BQMI (300 mg, 2.17 mmol, 1 equiv.) in absolute ethanol (50 mL). The reaction mixture was heated to reflux for 4 days. The formed precipitate was filtered on a sintered glass. The crude solid was washed with ethyl acetate, DCM, acetone, and Et_2_O to afford product 4 as a black solid (194 mg, 28% yield). M.P: >300 °C (decomposition). ^1^H NMR (400 MHz, DMSO-d*6*): δ = 10.42 (br s, 2H, NH), 7.04 (d, *^3^J* = 7.7 Hz, 4H, CH_Ar_), 6.54 (d, *^3^J* = 7.7 Hz, 4H, CH_Ar_), 5.87 (s, 1H, CH), 5.42 (br s, 4H, NH_2_), 5.11 (s, 1H, CH). ^13^C{^1^H} NMR (101 MHz, DMSO-d*6*): δ = 172.4 (C_quat_), 153.3 (C_quat_), 148.2 (C_quat_), 125.3 (CH), 124.6 (C_quat_), 113.7 (CH), 98.1 (CH), 84.9 (CH). IR (neat, cm^−1^): ν = 3448, 3417, 3167, 1629, 1604, 1540, 1507, 1473, 1437, 1348, 1282, 1239, 1201, 1167, 1131, 945, 906, 867, 821, 756, 710. HRMS (ESI+): *m/z* calculated for [M+H^+^]: 321.1346 (C_18_H_17_N_4_O_2_^+^), found: 321.1344.

### 2.6. OFET Preparation

**Figure 1:**
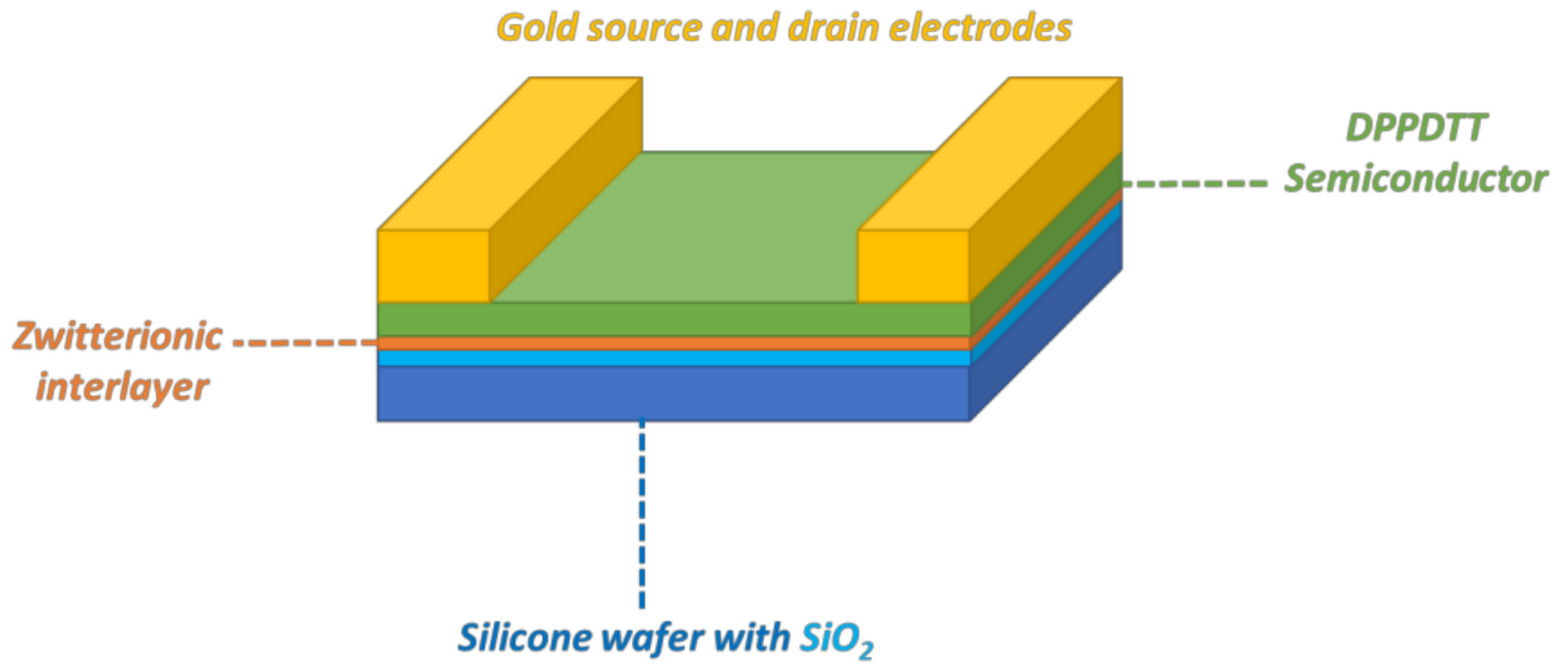
Scheme of an OFET with an Si gate electrode, SiO_2_ insulating layer, BQMI zwitterionic layer, DPPDTT semiconducting film, Au source, and drain electrodes.

The semiconducting material DPPDTT was dissolved in toluene to prepare a solution with a concentration of 10 mg per 1 mL. The BQMI molecules were dissolved in *N,N*-dimethylformamide (DMF) to obtain solutions with a concentration of 8 mmol L^−1^. Silicone substrates with a 300 nm SiO_2_ layer were cleaned in an ultrasonic bath (Bandelin electronic, Berlin, Germany)of isopropanol and acetone for 15 min. Next, the spin-coating method was used to produce BQMI layers (Spin-coater, 3D Nano, Kraków, Poland). The BQMI in DMF solutions were deposited onto the substrates and rotated with a velocity of 8000 RPM for 2 min. After cleansing and BQMI deposition, the substrates were transferred to a glove-box system with an inner nitrogen atmosphere. All semiconducting films were fabricated using the spin-coating technique: 200 µL of the solution was dispensed on the substrate, followed by spinning with a rotational velocity of 2000 RPM for 2 min. The film obtained as a result of the process had a thickness 60 nm. The prepared films were annealed at 90 °C for 1 h under a vacuum to remove the solvent residues. The gold source and drain electrodes were thermally evaporated through a shadow mask to a thickness of 60 nm (shown using a quartz crystal microbalance). As a result, OFETs were constructed in the bottom gate, with a top contact configuration (Figure 1). In all cases, the dimensions of the transistor channels were: width (W) 1 mm and length (L) 30 µm.

### 2.7. Measurements and Instruments

Nuclear magnetic resonance (NMR) spectra were recorded on a JEOL ECS400 NMR spectrometer (JEOL Ltd., Akishima, Japan) at room temperature. The NMR chemical shifts are given in ppm (δ) relative to Me4Si with solvent resonances used as internal standards (CDCl_3_: 7.26 ppm for ^1^H and 77.2 for ^13^C). Infra-red (IR) spectra were recorded on an Agilent Cary 630 FTIR (Agilent, Santa Clara, CA, USA) equipped with attenuated total reflectance (ATR) sampling. High-resolution mass spectrometry (HRMS) analyses were performed by the Spectropole center of chemical analysis at Aix-Marseille University using a QStar Elite (Applied Biosystems SCIEX, Toronto, ON, Canada) or SYNAPT G2 HDMS (Waters, Wilmslow, UK) spectrometer, each equipped with ESI or MALDI sources.

**Figure 2:**
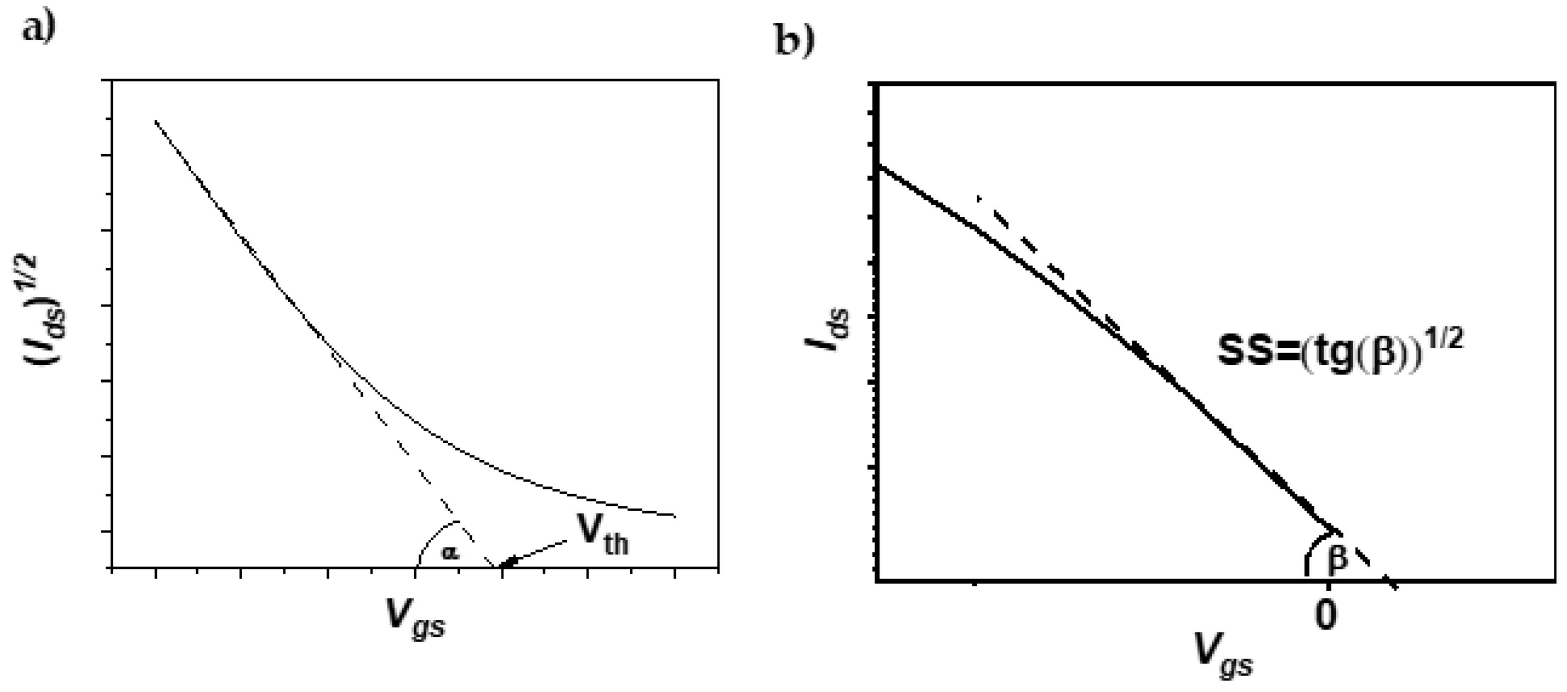
OFET transfer characteristics with a straight line intersecting the *V_gs_* axis at *V_th_* at the *α* angle fitted to the linear region of the curve (**a**); the subthreshold regime for the transfer characteristic with the line fitted to the transfer curve in half logarithmic scale (**b**).

Device characterization was performed under a nitrogen atmosphere inside the glove-box system (Blove-box line, Glove Box Technology Limited, Huntingdon, UK), using a Keithley 2634B Source Meter (Keithley Instruments, Cleveland, OH, USA) connected to a needle-probe station. Transfer and output characteristics were measure+d within a voltage regime from −70 V to 0 V. The mobilities of the charge carriers (µ_sat_) were calculated from the transfer characteristics in the saturation regime. To calculate mobility, a straight line was fitted to the linear region of the square root of the drain current in the saturation regime (I_DS_)^1/2^ (Figure 2a).

Charge carrier mobility was derived using the formula:

(1) $$\mu_{sat}=\;\frac{2\alpha^{2}\;L}{W\;C_{p}},$$

where *W* is the channel width, *L* is the channel length, *C_p_* is the capacitance per area unit of the gate dielectric (1.1 × 10^−4^ F m^−2^), and *α* is the slope of the fitted straight line. The charge carrier mobilities presented here are the average values obtained for 4–5 devices. The threshold voltage was estimated as the cross point of the fitted line and *V_gs_* axis. The subthreshold swing (*SS*) of the measured OFETs was determined as the reciprocal value of the slope of the fitting line to the subthreshold regime in the transfer curves of the OFETs (Figure 2b, Equation (2)).

(2) $$SS=\frac{dV}{d\left(log\left(I_{DS}\right)\right)}\;\;\mathrm{for}\;V_{GS}<V_{th}$$

The density of trap states at the interface (*N_it_*) was calculated using the equation [35]:

(3) $$N_{it}=\frac{C_{p}}{q^{2}}\left(\frac{qSS}{kT\left(ln10\right)}-1\right),$$

where *q* is the elementary charge and *T* is the temperature.

The ON/OFF ratio was calculated by dividing the Ids current for the highest *V_ds_* and *V_gs_* voltages by the *I_ds_* current for at the same *V_ds_* and *V_g_*_s_ = 0 V.

Electrostatic field homogeneity was tested using a scanning probe microscope (SPM) working in the electrostatic force microscope regime. Electrostatic field maps were obtained using a Nanosurf Flex atomic force microscope (AFM, Nanosurf GmbH, Langen, Germany) combined with a C3000 controller. The conductive tip and gate electrodes were polarized using a Keithley SourceMeter Unit 2612b (Keithley Instruments, Cleveland, OH, USA). The applied voltage was chosen experimentally and was established as 2 V (on the tip) and 10 V (on the gate). In this dynamic non-contact mode, the tip, while moving over the gate dielectric, is sensitive to the electrostatic field formed over the gate. Topography images were obtained using the atomic force microscope mode of the SPM. The data were processed using Gwyddion software (Version 2.58, Czech Metrology Institute, Brno, Czech Republic) [36]. Scanning electron microscopy (SEM) was performed using a Jeol JSM-7900F (JEOL Ltd., Akishima, Japan).

## 3. Results and Discussion

**Figure 3:**
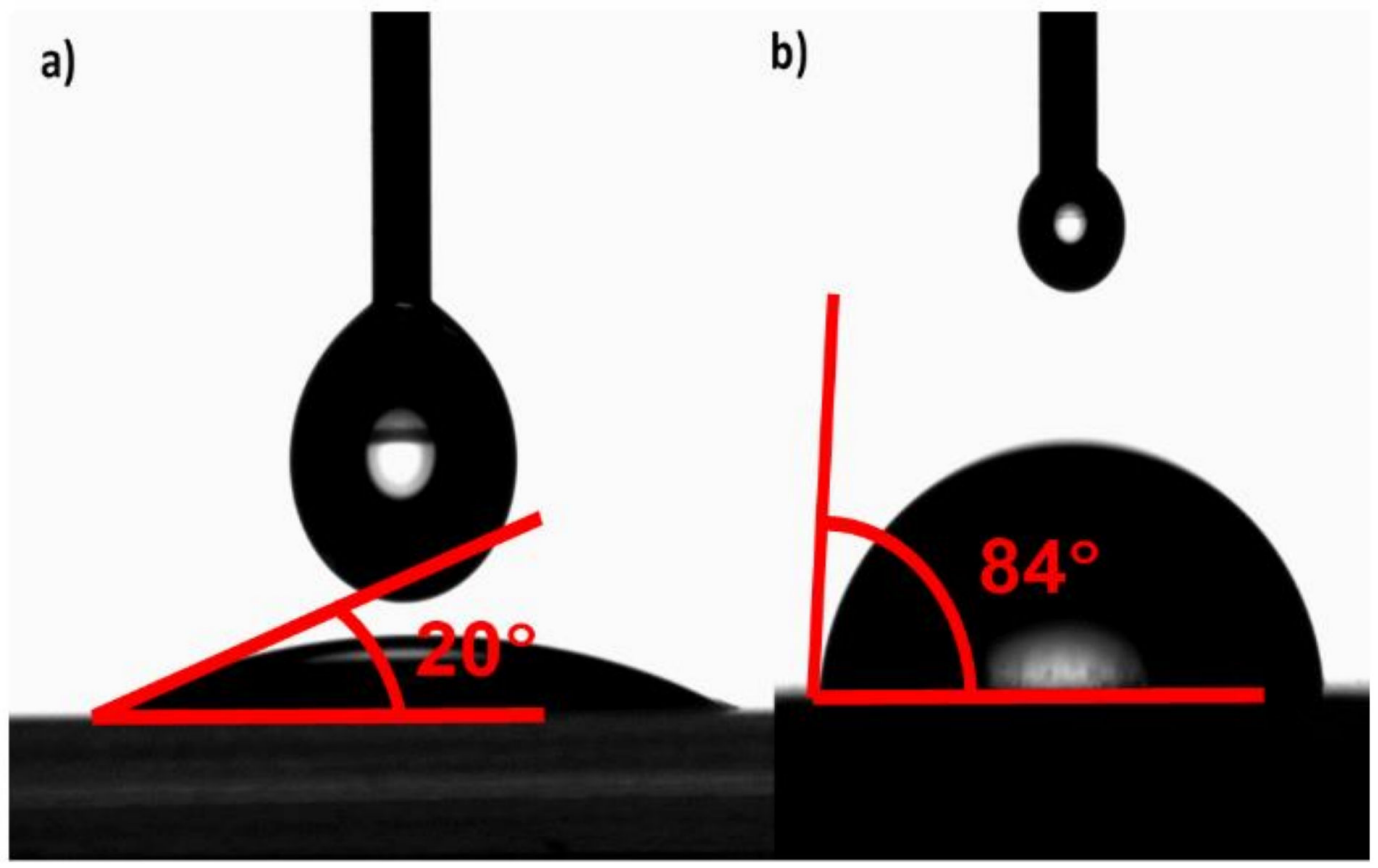
Droplet of water placed on the gate dielectric without a BQMI layer (**a**) and on a dielectric surface coated with BQMI compound **3** (**b**).

A series of *N*-substituted zwitterionic BQMIs compounds (**1–4**) was synthesized by straightforward transamination of commercially available 4,6-diaminoresorcinol dihydrochloride in the presence of an excess of amine (Scheme 1) [32]. The reaction proceeded faster in the presence of alkylamines (compounds **1–2**), whereas transamination using aniline derivatives required heating for several days to obtain compounds **3–4** [33,34]. The new lipophilic zwitterions **1–2** were isolated with yields of 60–73% after aqueous extraction followed by precipitation in pentane. They were characterized by ^1^H, ^13^C NMR and HRMS (see Materials Section and Supplementary Materials). To evaluate the hydrophobic character of the BQMI-modified SiO_2_ dielectric surfaces, the contact angle was measured. Figure 3 presents pictures of a droplet of pure deionized water placed on the bare dielectric (Figure 3a) and an oxide surface modified with compound **3** (Figure 3b). The contact angle for the bare oxide was around 20°, whereas on the modified surface it increased to 84°. This variation in the contact angle means that the surface coated with BQMI **3** was much more hydrophobic than bare silicon dioxide. Similar behavior was observed for all the tested BQMI compounds. The differences in contact angles between the compounds were in the range of +/– 5 degrees. Small differences may arise due to the optical method of determining CA. The hydrophobicity of the BQMI thin layer was stable in air. One week after deposition the contact angle was similar, at 82°. In general, BQMI zwitterions can be stored as powders under air conditions for months (even years) without undergoing any decomposition.

**Figure 4:**
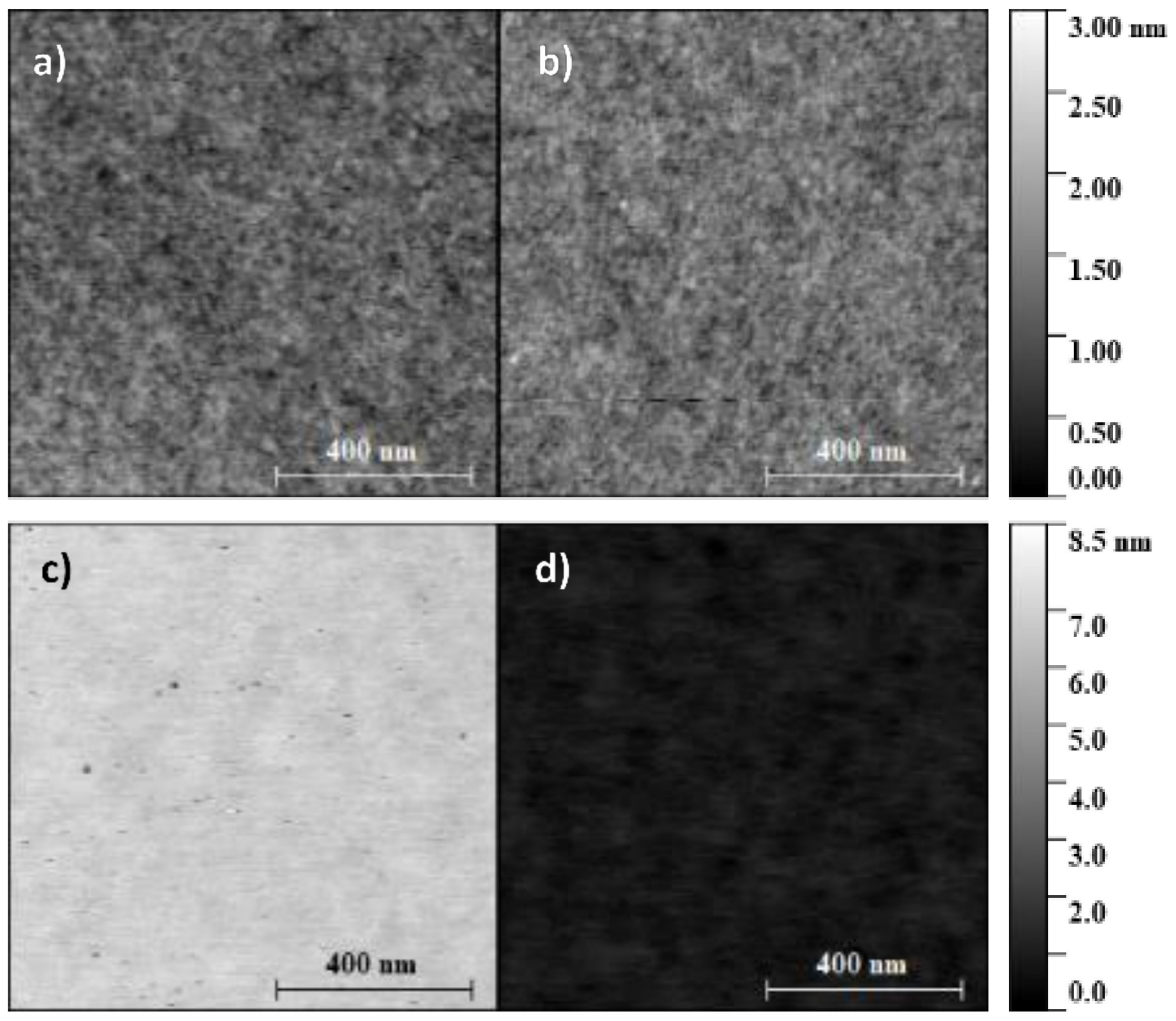
Atomic force microscope topography (**a**,**b**) and electrostatic force microscope images (**c**,**d**) of gate dielectrics without a BQMI layer (left side) and coated with BQMI **2** (right site).

The spin-coated BQMIs deposited directly on the SiO_2_ dielectric surface created thin interlayers. Scanning electron microscopy (SEM) was used to examine the cross-sections obtained for the zwitterion **3**-based layer deposited on a pure silicon substrate. However, the SEM pictures showed no visible BQMI layers, suggesting that their thickness was lower than 10 nm (see Supplementary Data). To analyze the quality of the BQMI-based interlayers, their topography was studied using AFM. Figure 4a,b present the topographies of the pure and coated dielectric surfaces. As can be seen, there were no significant differences in the topography of the layers. Electrostatic maps obtained for the uncoated dielectric surface and the dielectric surface coated with BQMI **2** are presented in Figure 4c,d, respectively. The electrostatic field over the non-coated surface exhibits sites (black dots) where the SPM cantilever was approached to the surface while scanning. This indicates a weak electrostatic field, caused by additional negative charges trapped under the surface (in the boron doped silicone gate electrode, as well as in the silicon oxide dielectric layer). The extra electrons may have come from the inhomogeneities created during silicon doping. After coating of the dielectric surface with BQMIs, the molecules create a uniform layer. Their shielding properties cause the presence of charge carrier traps that are not reflected in the electrostatic field. The electric field is homogeneous, and the absence of local distortion does not affect the charge carrier mobility in the OFETs. The other BQMI AFM images resemble those presented in Figure 4 (see Supplementary Materials).

**Figure 5:**
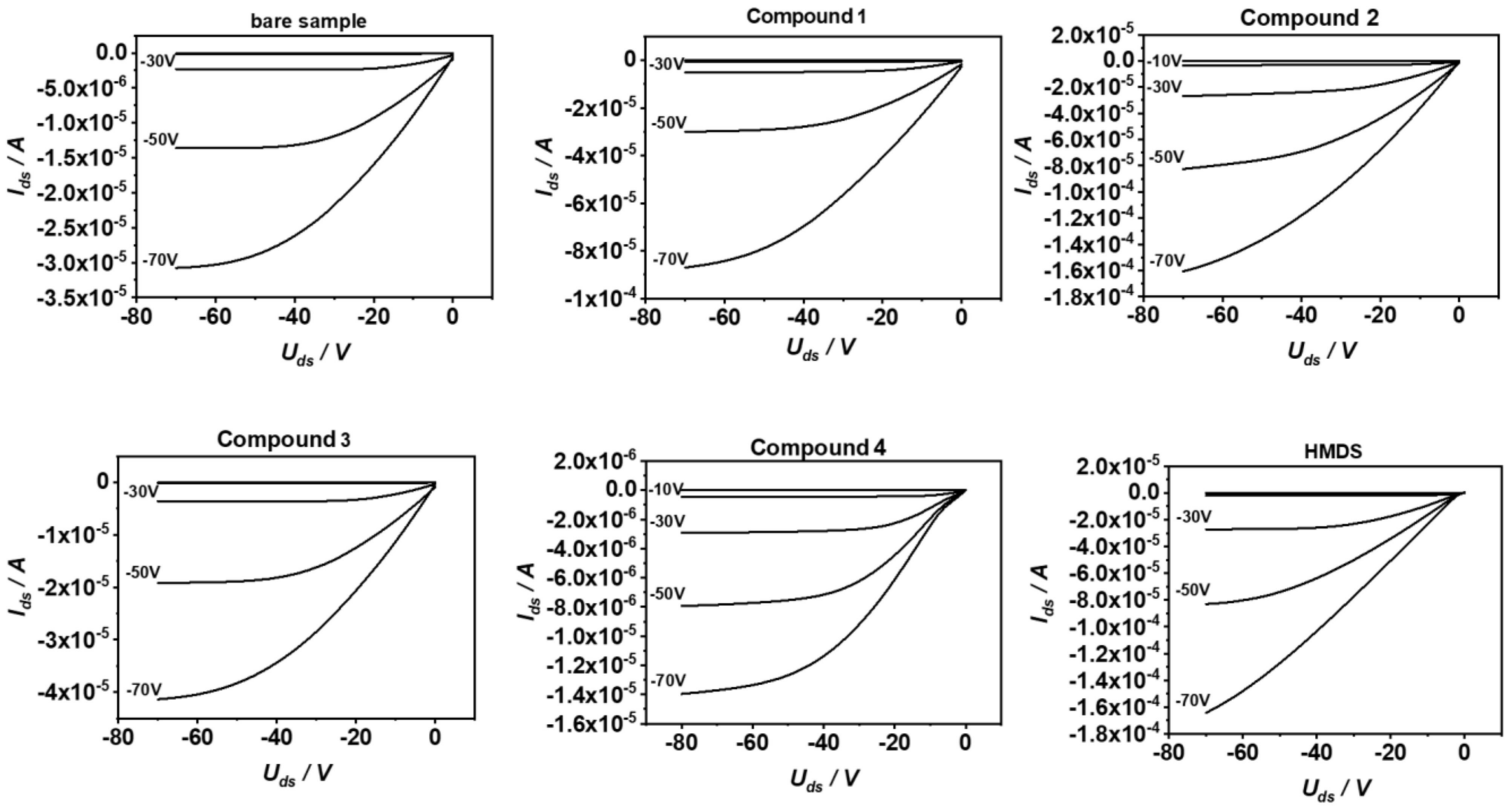
Output characteristics for transistors without a zwitterionic interlayer, with interlayers of BQMIs **1–4**, and with an HMDS interlayer.

**Figure 6:**
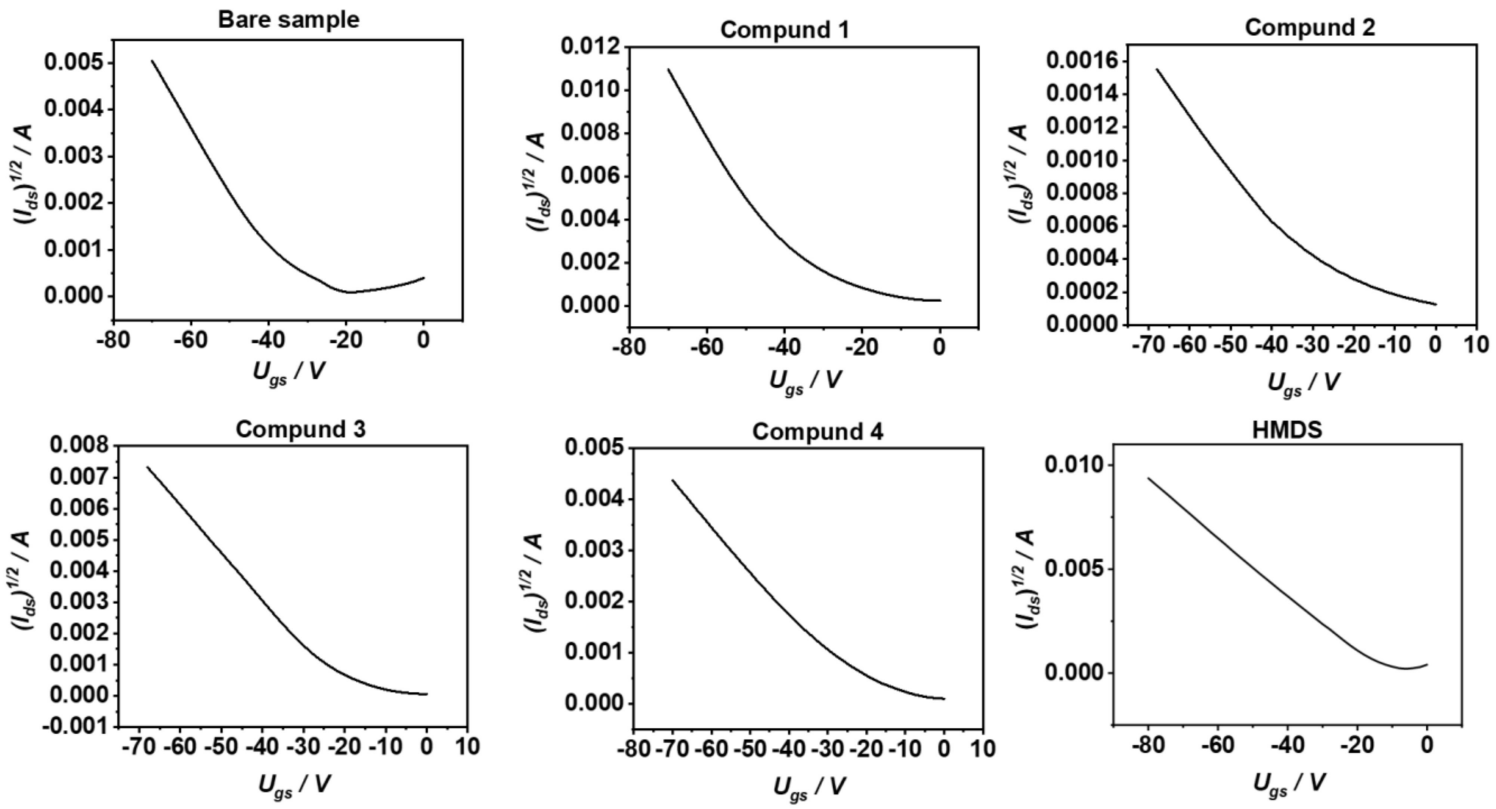
Transfer characteristics for transistors without a zwitterionic interlayer, with interlayers of BQMIs **1–4**, and with an HMDS interlayer.

To estimate the influence of the other derivatives of zwitterionic interlayers on the performance of the OFETs, a series of BQMI-based devices was fabricated with four different compounds (**1–4**). The morphology of the BQMI layers was examined by means of AFM. The roughness of the layers did not exceed 2 nm, and their surface topographies were similar. Transistors without an interlayer and with an HDMS interlayer were elaborated as references, as the mechanism of HDMS operation is similar to the mechanism of zwitterion operation, being mainly based on a reduction in the number of trapping sites. The measured output current voltage characteristics of the OFETs are presented in Figure 5. The transfer curves are presented in Figure 6.

All measured characteristics were consistent with previously reported data for p-type OFETs [37]. In the samples with a BQMI interlayer, the value of the OFF current reduced from 1.6 × 10^−7^ A for the bare sample to 6.2 × 10^−8^ A for BQMI compound **1**, to 8.7 × 10^−8^ A for BQMI compound **2**, to 2.9 × 10^−8^ A for BQMI compound **3**, and to 5.6 × 10^−9^ for BQMI compound **4**. 

**Figure 7:**
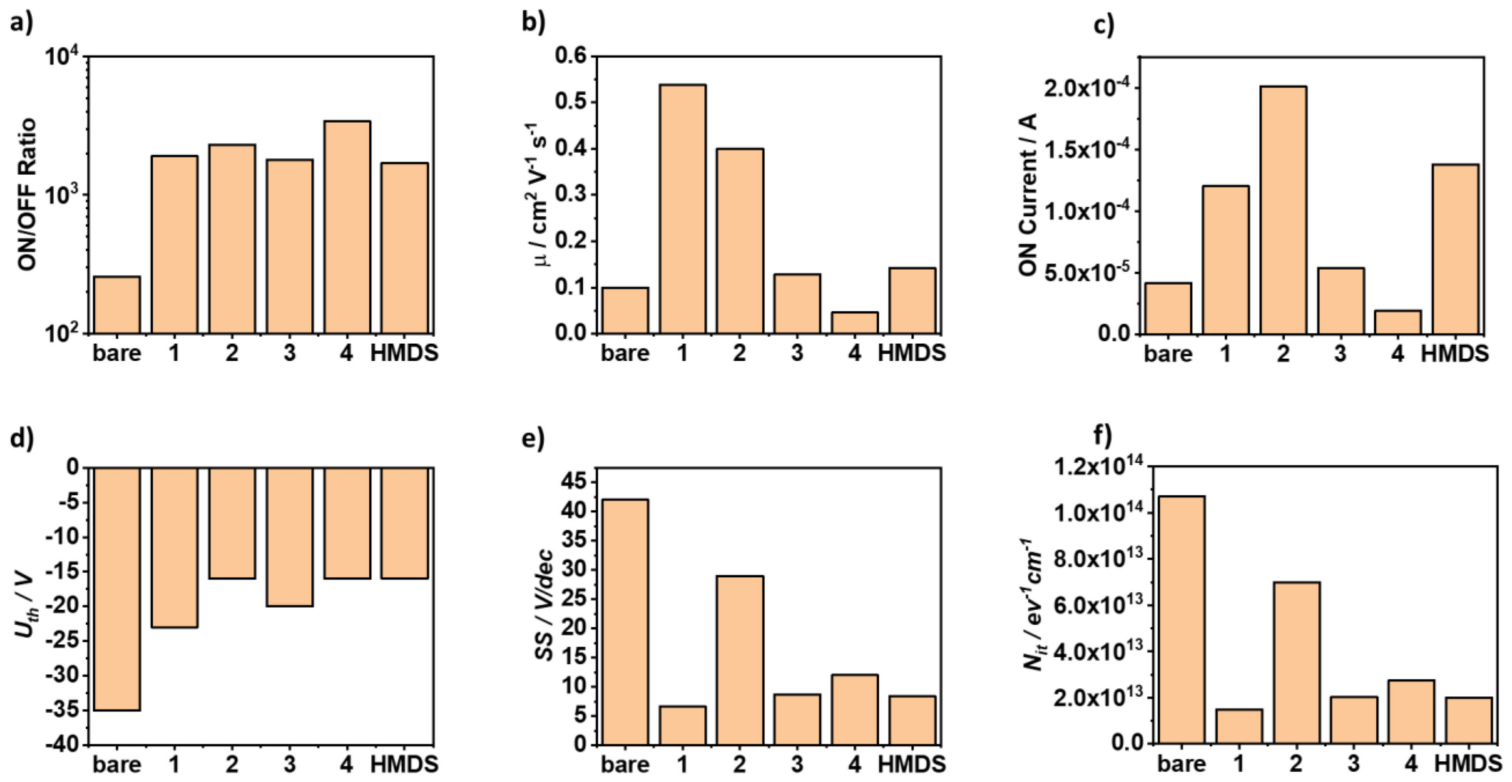
Bar charts comparing the ON/OFF ratio (**a**), charge carrier mobility (**b**), ON current (**c**), threshold voltage (**d**), subthreshold swing (**e**), and density of the interface traps (**f**) determined for selected OFETs with bare oxide BQMI compounds **1–4** or HMDS dielectric surface modification.

By comparison, the OFF current of the sample with an HMDS interlayer was 8 × 10^−8^ A. Figure 7 compares the relevant OFETs parameters of selected field effect transistors, including charge carrier mobility, ON current, and the ON/OFF ratio.

Charge carrier mobility was higher in the devices coated with BQMI compounds **1–3** compared to the bare sample. The introduction of the interlayer based on compound **4** resulted in poor charge carrier mobility, which may be explained by the relatively low solubility of this compound in organic solvents, including DMF solution. This necessitated the lowering of the concentration of DMF solution, which resulted in the creation of discontinuous films. Importantly, the charge carrier mobility values calculated for the transistors containing interlayers based on zwitterions **1** and **2** were higher (respectively: 5.4 × 10^−1^ and 4.0 × 10^−1^ cm^2^ V^−1^ s^−1^) than the mobility values calculated for the sample with an HMDS interlayer (1.3 × 10^−1^ cm^2^ V^−1^ s^−1^). BQMI derivatives **1** and **2** both have relatively long aliphatic side groups, and their flexibility may influence the packing density of the molecules on the dielectric surface, improving the smoothness of the interlayers. Bao and co-workers reported higher charge carrier mobility in devices with silane-coated dielectric layers with dense and crystalline OTS surface-modified layers than with disordered OTS [38]. The presence of such densely packaged interlayers may reduce the charge carrier trapping effect at the dielectric–semiconductor interface.

**Figure 8:**
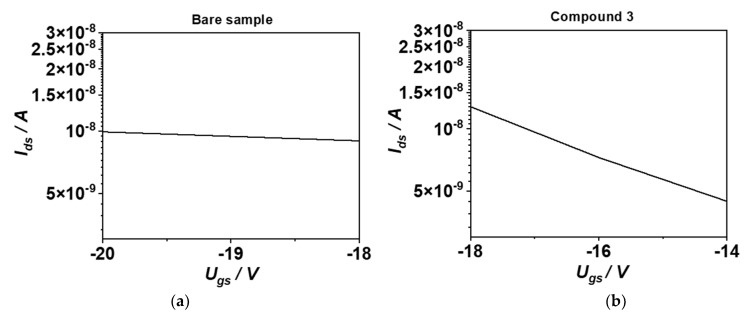
Subthreshold regime of transfer curves for transistors on silicon dioxide without a BQMI layer (**a**) and coated with compound **3** (**b**).

To verify whether this was the case, the density of the N_it_ traps was calculated using Equation (3) and SS taken from the OFET transfer characteristics (Figure 8). For OFETs with the bare oxide, the N_it_ value was around 10^14^ eV^−1^ cm^−2^, and for the transistors with a dielectric surface modified by BQMIs or HMDS the N_it_ was nearly 2 × 10^13^ eV^−1^ cm^−2^.

In the case of transistors with a modified SiO_2_ surface, the reduction in the density of the traps contributed to an increase in the drain current flow.

## 4. Conclusions

Charge carriers are accumulated and transported in the dielectric–OSC interface. Therefore, its quality is crucial for OFET performance. A proper understanding and description of the interface is crucial to improving the efficiency of OFETs. The experimental work reported in this study demonstrates a new and easier method of modifying the silicon oxide layer, which allows for improvements in several parameters compared to transistors without modified dielectric surfaces. Our method is both less costly and less time-consuming than other commonly used modification techniques, such as coating dielectric layers on HMDS.

The zwitterions with aromatic side groups (**3**–**4**) were characterized by weaker solubility in DMF in comparison to their aliphatic analogues. Their incorporation into the OFET structure as interlayers did not lead to improved charge carrier mobility or increase the current flowing through the transistor channel. In contrast, the coating of bare SiO_2_ with N-alkyl zwitterionic quinones caused changes to the water contact angle, as observed for compound **3**. The modified surface was characterized by a contact angle value of around 84°, compared to 20° before modification. This presumably resulted in better ordering of the DPPDTT molecules in the semiconductor layer and improved the features of the OFET. The charge carrier mobility increased from 0.1 cm^2^ V^−1^ s^−1^ for the bare sample to 0.55 cm^2^ V^−1^ s^−1^ for the sample with the incorporation of zwitterion **1** as an interlayer.

A similar effect was observed for zwitterion **2**. Both compounds are equipped with aliphatic side groups, allowing for higher packing density on the dielectric surface and improvement of the smoothness of the interlayers. Nevertheless, the transistors with zwitterion **2** were characterized by a relatively high value for SS, showing that the growth dynamic of the current in the subthreshold regime was low. This might be connected with the relatively high density of the traps at the semiconductor–dielectric interface. The shape of the output curves was a closer fit to the model transistor [39] in the case of the samples modified by BQMIs as compared to the HMDS-treated sample. The saturation region of the IV output characteristic began at a lower drain voltage, and its linear regime was shorter. The modification of the SiO_2_ surface with zwitterion **1** resulted in a decrease in the density of the surface charge carrier traps, by about one order of magnitude. This was related to the absence of disturbances in the electrostatic field near the modified surface, as confirmed by electrostatic force microscopy. This work opens new perspectives for the use of BQMI zwitterions in organic electronics, enabling highly versatile synthesis and fine-tuning of properties depending on the target applications.

## Data Availability

The data presented in this study are available on request from the corresponding authors.

## Supplementary Materials

The following are available online at https://www.mdpi.com/article/10.3390/polym13101567/s1. Figure S1: ^1^H NMR (400 MHz, CDCl_3_) of compound **1**. Figure S2: ^13^C NMR (100 MHz, CDCl_3_) of compound **1**. Figure S3: 1H NMR (400 MHz, CDCl3) of compound **2**. Figure S4: ^13^C NMR (100 MHz, CDCl_3_) of compound 2. Figure S5: Infrared spectrum of compound 1. Figure S6: Infrared spectrum of compound **2**. Figure S7: HRMS spectrum of compound **1**. Figure S8: HRMS spectrum of compound **2**. Figure S9: SEM picture of the cross-section of the BQMI 3 spin-coated layer on a silicon wafer. Figure S10: AFM picture of the BQMI **1** spin-coated layer on a silicon wafer. Figure S11: AFM picture of the BQMI **3** spin-coated layer on a silicon wafer. Figure S12: AFM picture of the BQMI **4** spin-coated layer on a silicon wafer.
