# Supplementary material for: The Quinonoid Zwitterion Interlayer for the Improvement of Charge Carrier Mobility in Organic Field-Effect Transistors
Source: Polymers (Basel). 2021 May 13;13(10):1567. doi: 10.3390/polym13101567 (PMC8153292; doi:10.3390/polym13101567)
Supplement: Supplementary file 1 [file polymers-13-01567-s001.zip › polymers-1210600-supplementary.pdf]

polymers

MDPI

Supplementary Materials

Quinonoid Zwitterions Interlayer for the Improvement of Charge Carriers Mobility in Organic Field Effect Transistors

Adam Luczak [1], Angélina Torres Ruiz [2], Simon Pascal [2], Adrian Adamski [1,3], Jaroslaw Jung [1], Beata Luszczynska [1],* and Olivier Siri [2],*

[1] Lodz University of Technology, Faculty of Chemistry, Department of Molecular Physics, Zeromskiego 116, 90-924 Lodz, Poland.

[2] Aix Marseille Univ, CNRS, CINaM, UMR 7325, Campus de Luminy, 13288 Marseille cedex 09, France.

[3] Department of Experimental Physics, Faculty of Fundamental Problems of Technology, Wroclaw Uni-versity of Science and Technology, wyb. Stanislawa Wyspianskiego 27, 50-370 Wroclaw, Poland.

* Correspondence: beata.luszczynska@p.lodz.pl (B.L.); olivier.siri@univ-amu.fr (O.S.)

TABLE OF CONTENT

I. NMR SPECTRA 2

II. INFRARED SPECTROSCOPY 4

III. MASS SPECTROMETRY 5

IV. SCANNING ELECTRON MICROSCOPY 6

V. ATOMIC FORCE MICROSCOPY 7

I. NMR SPECTRA

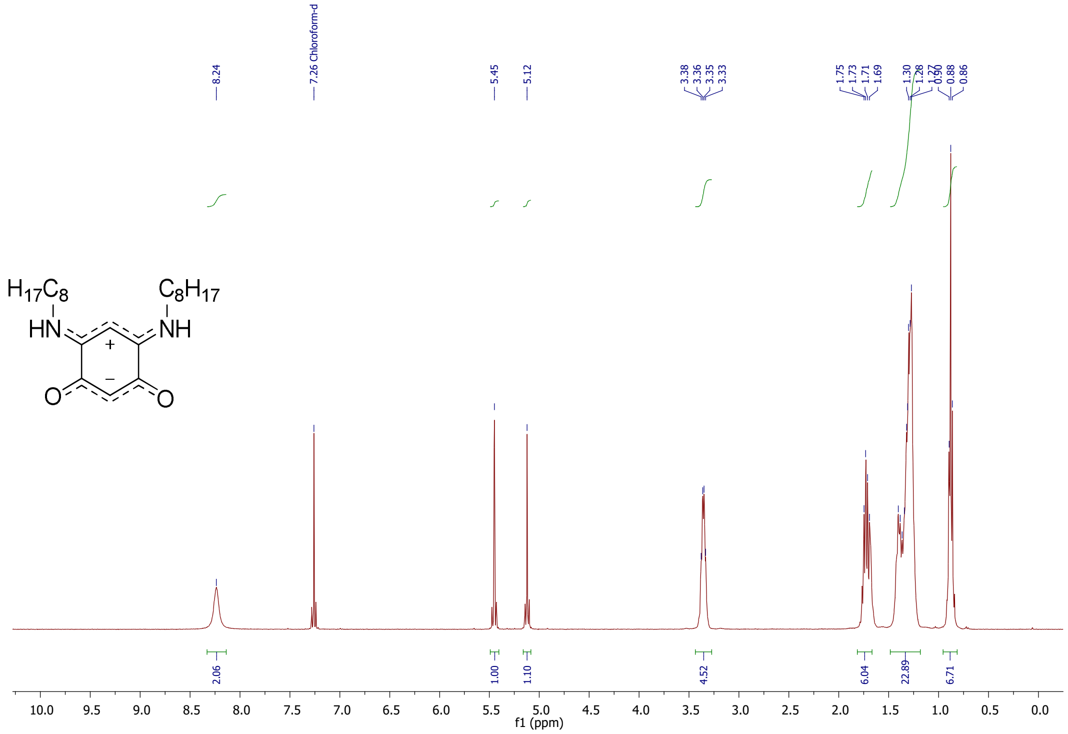

Figure S 1. ^{1}H NMR (400 MHz, $CDCl_3$) of compound **1**.

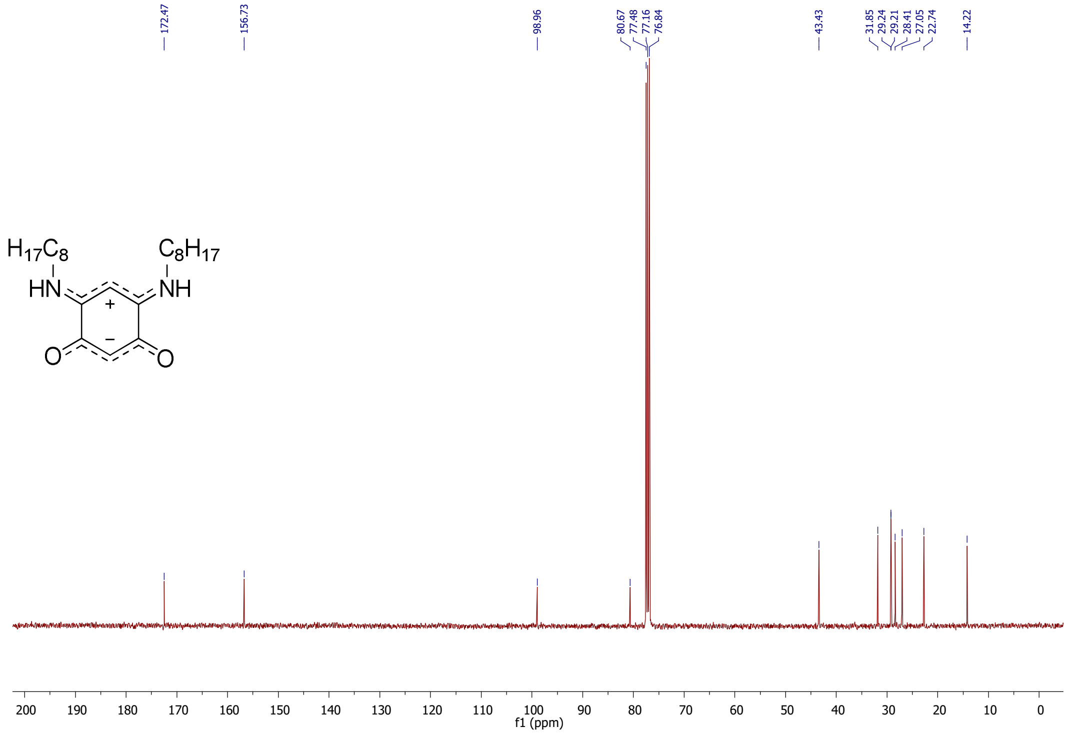

Figure S 2. ^{13}C NMR (100 MHz, $CDCl_3$) of compound **1**.

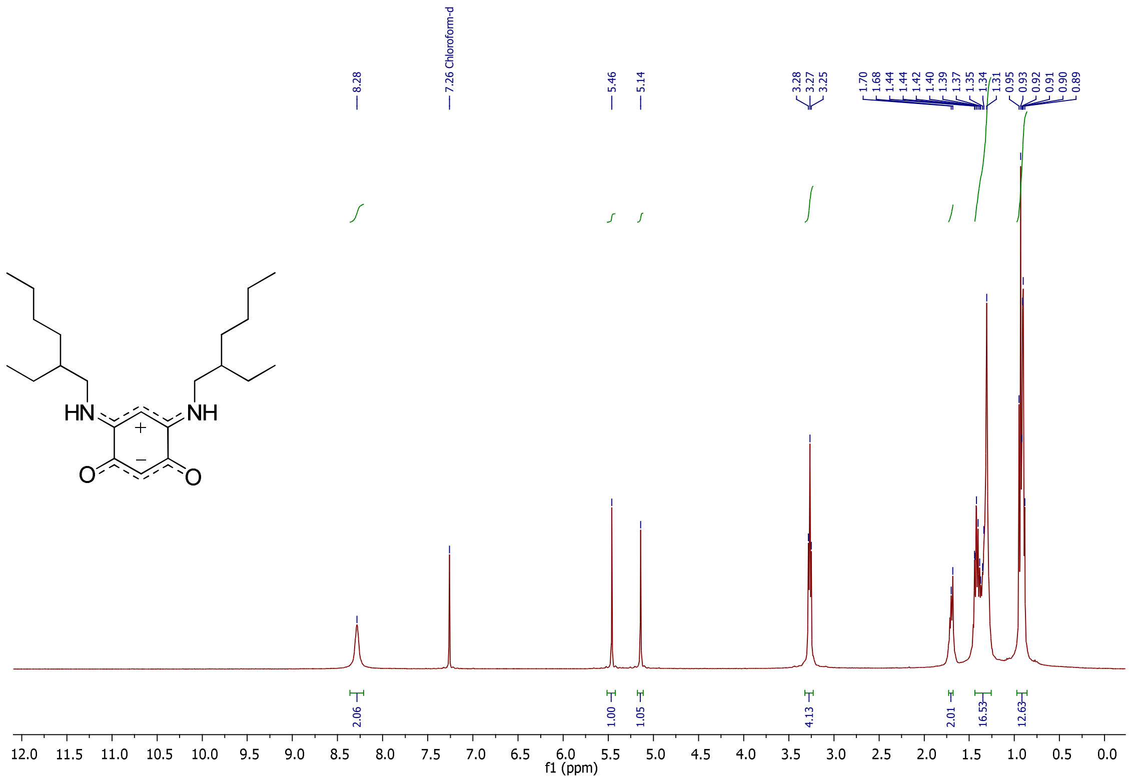

Figure S 3. ^{1}H NMR (400 MHz, $CDCl_3$) of compound **2**.

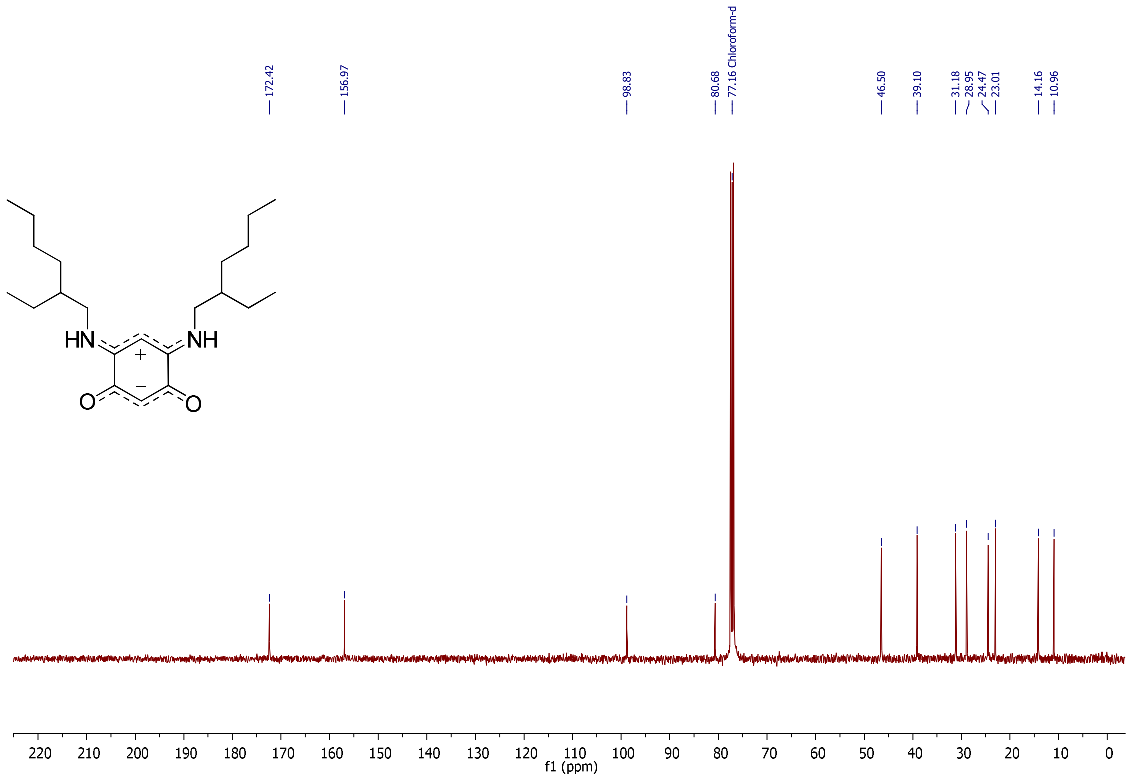

Figure S 4. ^{13}C NMR (100 MHz, $CDCl_3$) of compound **2**.

II. INFRARED SPECTROSCOPY

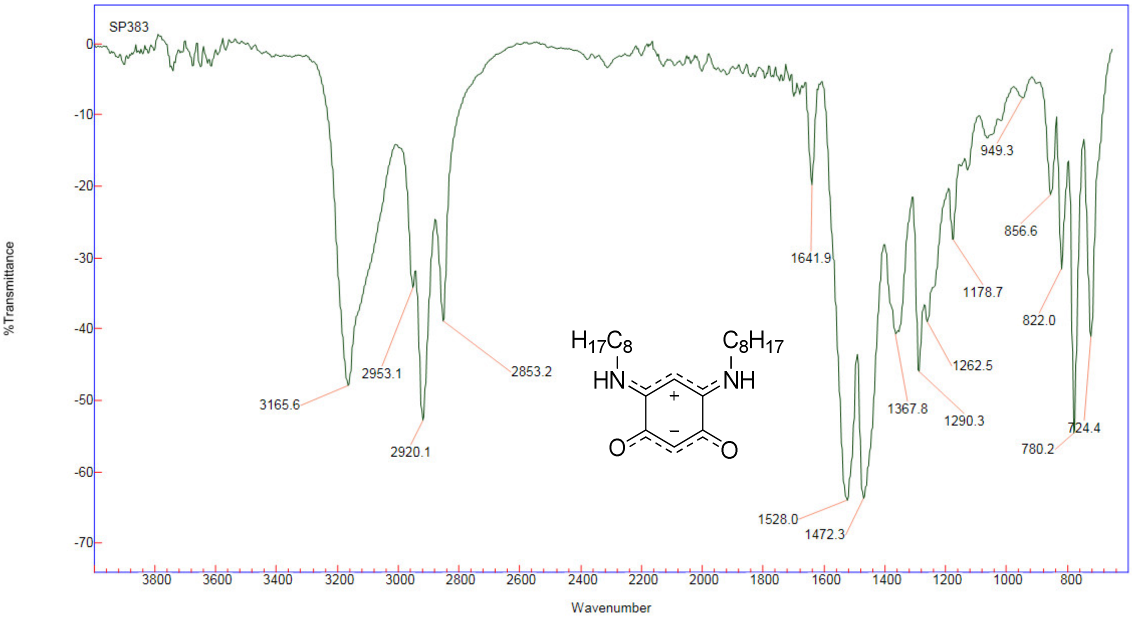

Figure S 5. Infrared spectrum of compound **1**.

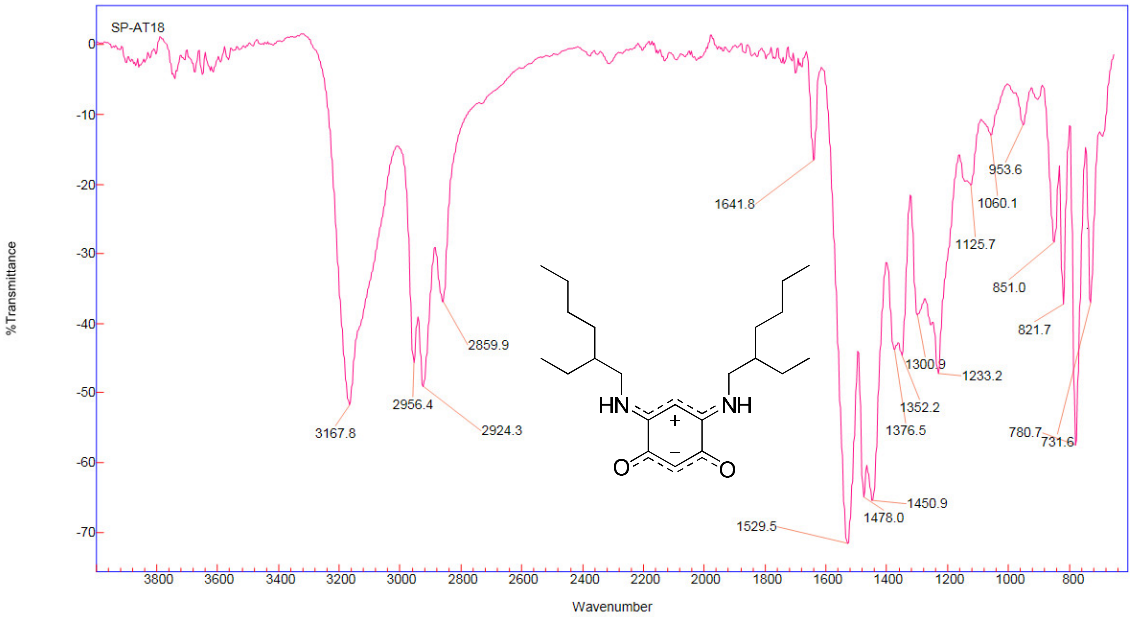

Figure S 6. Infrared spectrum of compound **2**.

III. MASS SPECTROMETRY

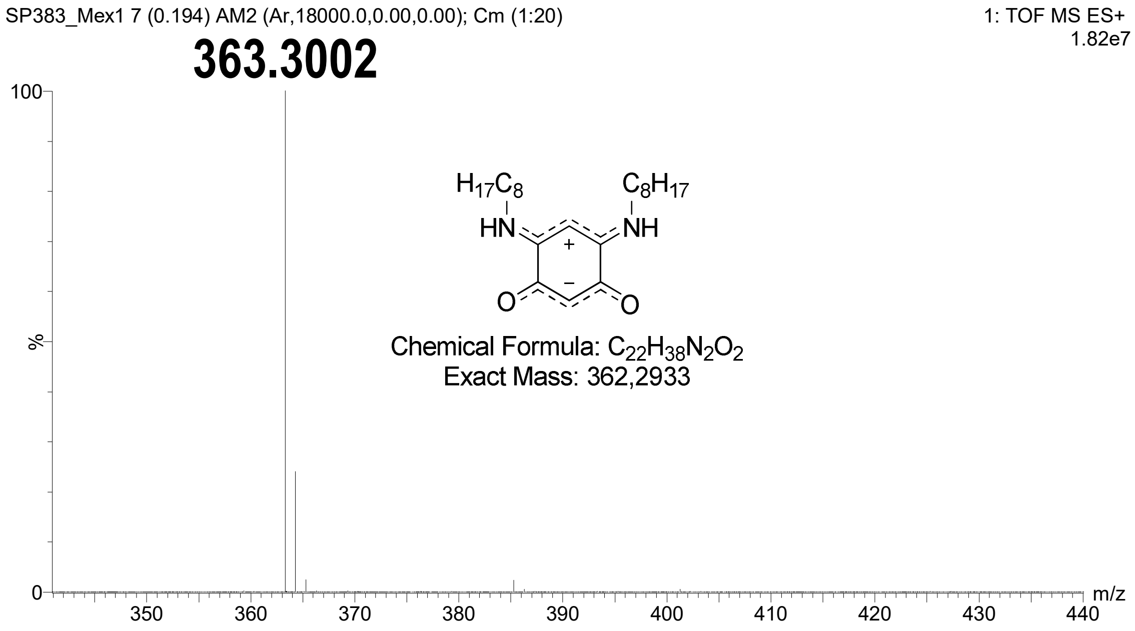

Figure S 7. HRMS spectrum of compound **1**.

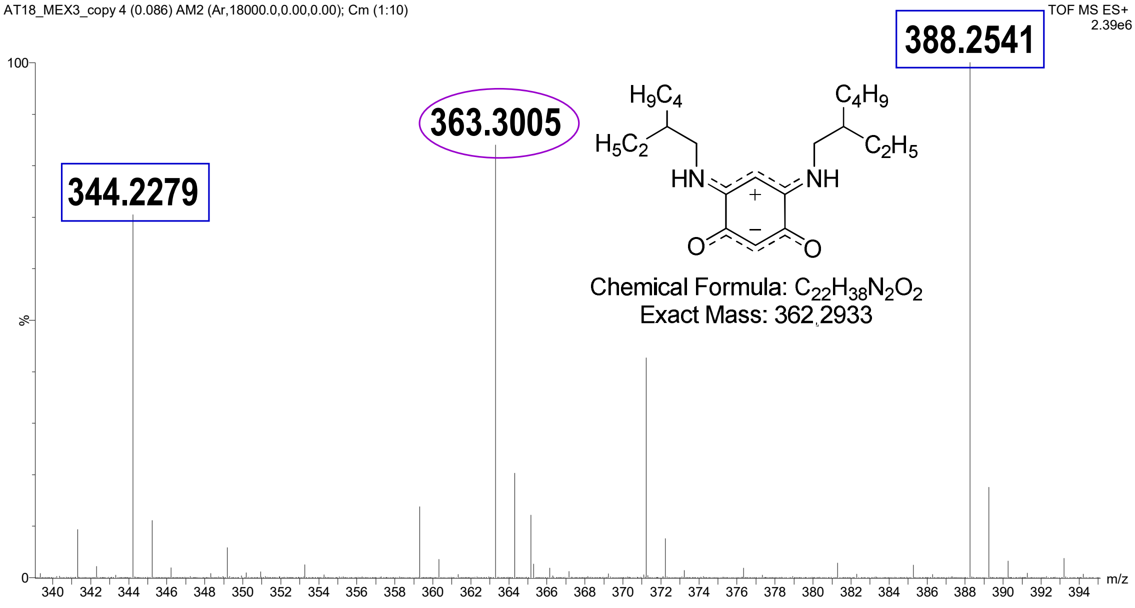

Figure S 8. HRMS spectrum of compound **2**. Target ion is detected at m/z 363.3005 and calibration peaks are observed at m/z 344.2279 and m/z 388.2541.

IV. SCANNING ELECTRON MICROSCOPY

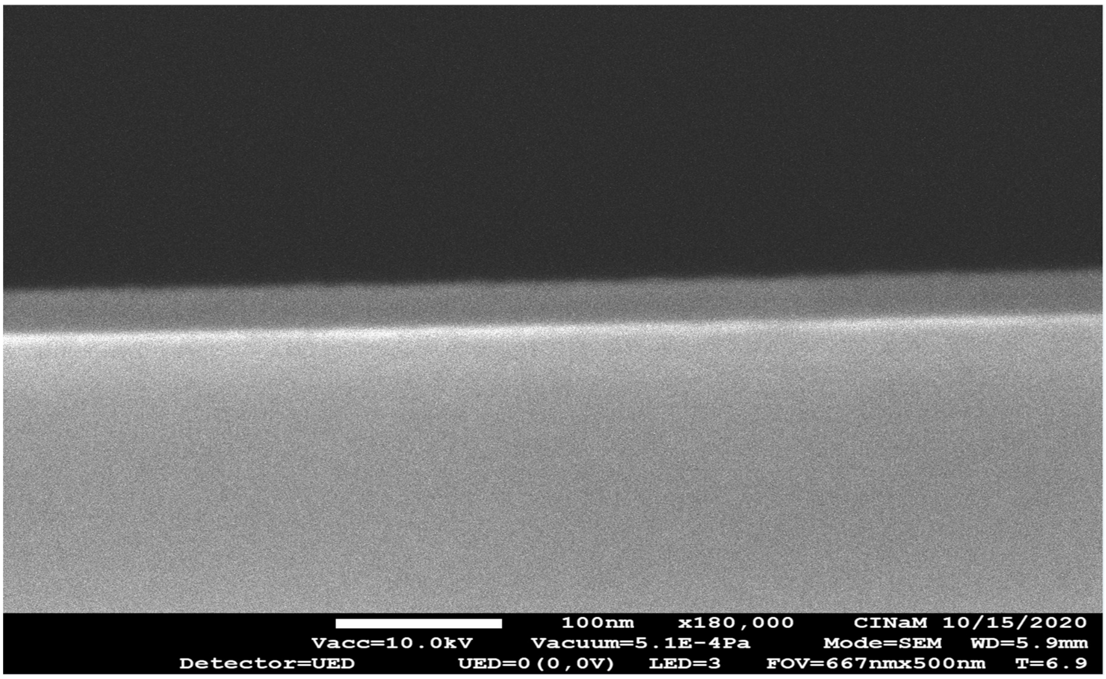

Figure S 9. SEM picture of the cross-section of the BQMI **3,** spin-coated layer on a silicon wafer.

V. ATOMIC FORCE MICROSCOPY

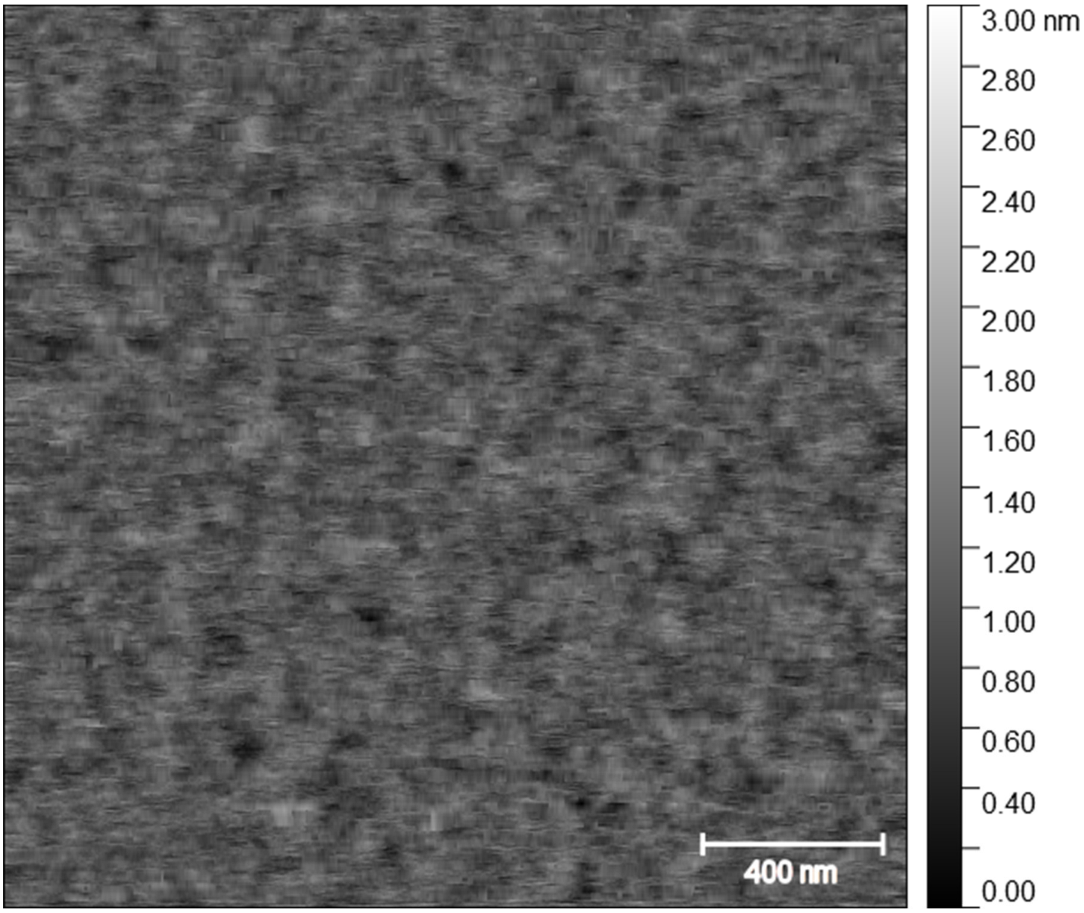

Figure S 10. AFM picture of the BQMI **1** spin-coated layer on a silicon wafer.

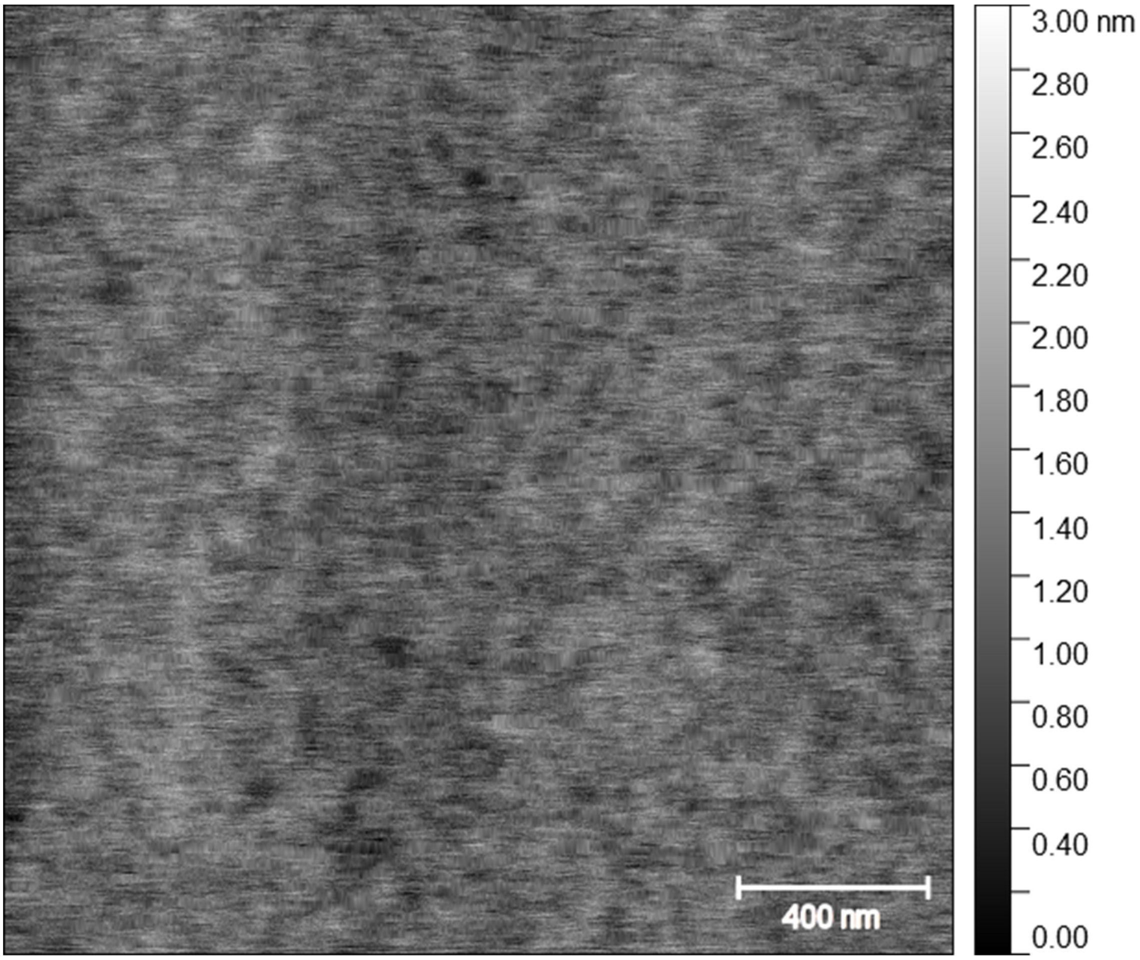

Figure S 11. AFM picture of the BQMI **3** spin-coated layer on a silicon wafer.

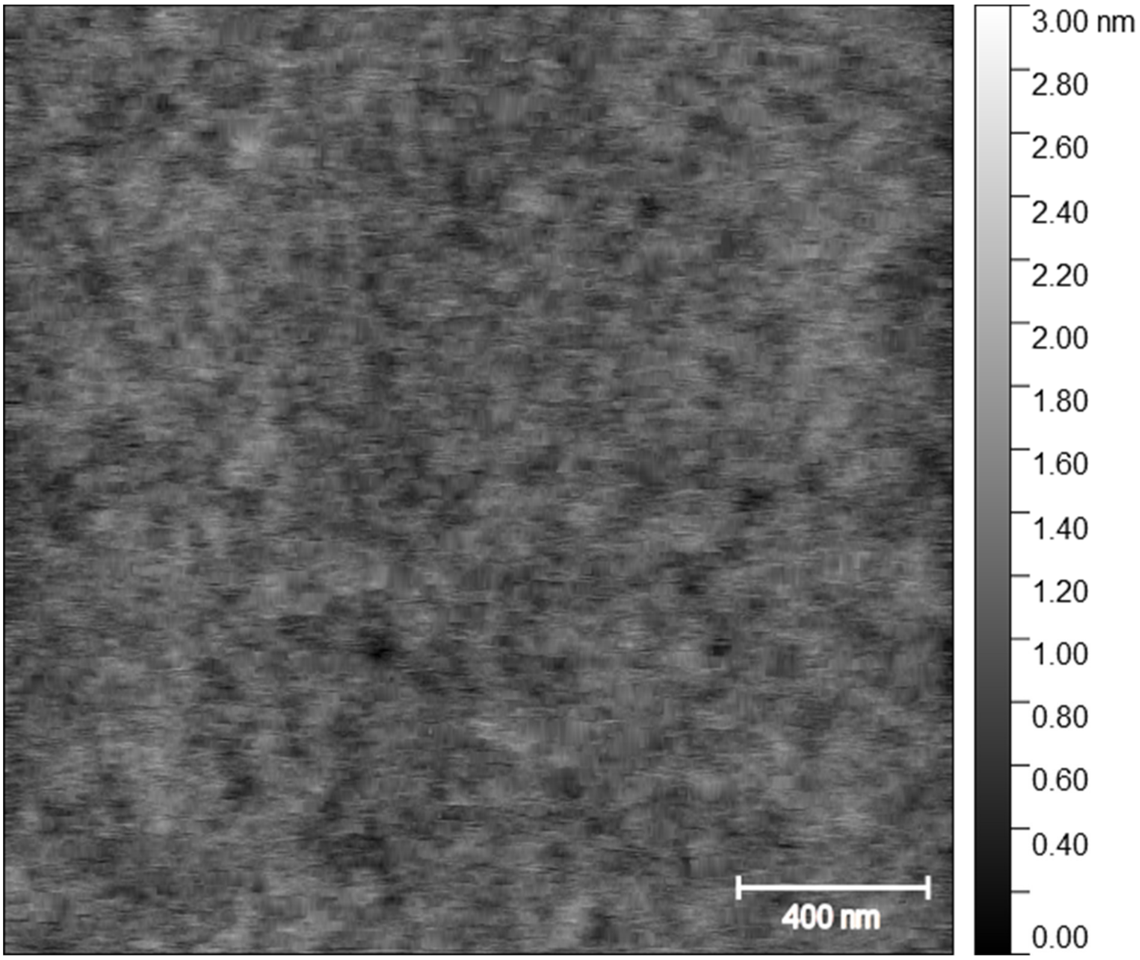

Figure S 12. AFM picture of the BQMI **4** spin-coated layer on a silicon wafer.
